# CRK9 contributes to regulation of mitosis and cytokinesis in the procyclic form of *Trypanosoma brucei*

**DOI:** 10.1186/1471-2121-10-68

**Published:** 2009-09-21

**Authors:** Stephane Gourguechon, Ching C Wang

**Affiliations:** 1 Department of Pharmaceutical Chemistry, University of California, San Francisco, CA 94158-2280, USA

## Abstract

**Background:**

The *Trypanosoma brucei *cell cycle is regulated by combinations of cyclin/CRKs (cdc2 related kinases). Recently, two additional cyclins (CYC10, CYC11) and six new CRK (CRK7-12) homologues were identified in the *T. brucei *genome database [1,2].

**Results:**

Individual RNAi knockdowns of these new proteins in the procyclic form of *T. brucei *showed no apparent phenotype except for the CRK9 depletion, which enriched the cells in G2/M phase. But a similar CRK9 knockdown in the bloodstream form caused no apparent phenotype. CRK9 lacks the typical PSTAIRE motif for cyclin binding and the phenylalanine "gatekeeper" but binds to cyclin B2 *in vitro *and localizes to the nucleus in both forms of *T. brucei*. CRK9-depleted procyclic-form generated no detectable anucleate cells, suggesting an inhibition of cytokinesis by CRK9 depletion as well. The knockdown enriched cells with one nucleus, one kinetoplast and two closely associated basal bodies with an average distance of 1.08 mm in between, which was shorter than the control value of 1.36 μm, and the cells became morphologically deformed and rounded with time.

**Conclusion:**

CRK9 may play a role in mediating the segregation between the two kinetoplast/basal body pairs prior to cytokinetic initiation. Since such a segregation over a relatively significant distance is essential for cytokinetic initiation only in the procyclic but may not be in the bloodstream form, CRK9 could be specifically involved in regulating cytokinetic initiation in the procyclic form of *T. brucei*.

## Background

Regulation of eukaryotic cell cycle is mediated primarily by signaling cascades orchestrated by the activities of protein kinases. The most extensively studied class of protein kinases is the cyclin-dependent kinases (Cdks) such as the prototypic Cdc28 regulating the cell cycle in budding yeast [3-10]. Cdk activity requires binding to a specific cyclin and thus is regulated by the periodic synthesis and degradation of cyclins [3,11,12].

*Trypanosoma brucei *is a parasitic protozoan and the causative agent of sleeping sickness, a devastating disease responsible for considerable morbidity and mortality in sub-Saharan Africa. It is also considered a deeply branched eukaryote with many unique biological features [13,14]. Individual *T. brucei *cells possess a single mitochondrion whose genome is arranged in a disc-like structure, the kinetoplast, which is associated with a basal body across mitochondrial membrane [15-17]. The basal body serves as a microtubule organizing center, the origin of flagellum, and provides the driving force for kinetoplast segregation prior to cytokinesis [16,18]. *T. brucei *cells transit through several stages in its life cycle to three major replicative phases: the procyclic stage growing in the tsetse fly midgut, and the bloodstream form proliferating in the mammalian host bloodstream [19,20]. Both of these stages can be cultivated *in vitro*. A third replicative stage in the epimastigote form in the salivary gland of tsetse fly has not yet been adapted to *in vitro *cultivation and is thus relatively un-explored. There are many crucial differences between the procyclic and bloodstream forms, the most obvious one being the position of the kinetoplast/basal body complex; it is midway between the posterior end of the cell and the nucleus in procyclic cells, whereas, in bloodstream form cells, it is localized at the posterior end [16,18,21]. Upon the replication of kinetoplast/basal body and nucleus in the procyclic form, the newly formed kinetoplast/basal body migrates to the posterior end of the cell, whereas the newly formed nucleus is moved in between the two segregated kinetplasts/basal bodies prior to the initiation of cytokinesis [22]. But in the bloodstream form, the two kinetoplasts/basal bodies remain at the posterior end after replication and are separated by less than 2.5 μm (Tyler et al., 2001), whereas the two nuclei stay in the mid-portion of the cell. There is thus no apparent need for migration of these organelles over significant distances to signal the beginning of cytokinesis in bloodstream form [16,20,22]. It is not clear whether even the short distance of less than 2.5 μm between the two kinetoplast/basal body pairs is needed for initiating cytokinesis in the bloodstream form.

*T. brucei *has several conserved homologues of cyclins and cyclin-dependent kinases termed cdc2-related kinases (CRKs) [23-27] some of which function analogously to those in other eukaryotes [24,26,27]. Recent studies have identified the complex of cyclin E1 and CRK1 in regulating G1/S transition and the complexes of cyclin E1 and CRK3 as well as cyclin B2 and CRK3 in controlling the passage of G2/M boundary during the cell cycle progression [25,27,28]. CRK2 was found to complex with cyclin E1 in controlling the cellular morphogenesis during G1/S transition in the procyclic form but not in the bloodstream form [26,28], whereas cyclins E2, E3, E4, B1 and B3 as well as CRKs 2, 4 and 6 have not yet been identified with an apparent function as their knockdowns by RNA interference (RNAi) showed no apparent phenotype in the procyclic form of *T. brucei *[24,26-28]. Recently, several new homologues of cyclins (10 and 11) and CRKs (CRK7-12) were identified in the *T. brucei *genomic database using a bioinformatics approach [1,2], but there has not yet been any function assigned to these newly identified proteins.

In the present study, we used RNAi to investigate the potential functions of these newly identified homologues in the procyclic form of *T. brucei *and found them playing no significant role in regulating cell proliferation except for CRK9, which was found essential for cell growth in the procyclic-form but not in the bloodstream-form cells. Depletion of CRK9 enriches the procyclic cells in G2/M phase with two kinetoplast/basal body pairs incapable of sufficient separation from each other for cytokinetic initiation [29]. An accompanying drastic morphological alteration turns the cells into a round shape. CRK9 is apparently playing a critical role in regulating both mitosis and cytokinesis in the procyclic cells.

## Methods

### DNA constructs

Cloning of DNA fragments used standard methods [30] and constructs were verified by DNA sequencing. For RNAi constructs, 300-500 nucleotide fragments of target genes were amplified by PCR, using primers that incorporated XhoI and HindIII sites at opposite ends of the DNA fragment. Fragments thus digested with XhoI/HindIII were inserted into the pZJM vector, linearized with NotI and introduced into *T. brucei *for homologous recombination in the rRNA intergenic regions for RNAi of the targeted mRNA. For expression of recombinant proteins in transformed *Escherichia coli*, a full-length gene was inserted as a BamHI/SalI fragment into the pGEX6P3 vector (GE Healthcare). For expressing tagged protein at the endogenous level in *T. brucei*, the encoding gene was inserted as a HindIII/SalI fragment into the HindIII/XhoI sites of pLew-3HA vector to create an open reading frame of C-terminally 3xHA (hemagluttinin) tagged protein. An ApaI/EcoRI fragment of the DNA containing part of the CRK9 gene fused with a 3xHA tag, was then inserted into the pC-PTP-BLA vector, linearized with XhoI and transfected into *T. brucei *for homologous recombination [31] and endogenous level expression of the C-terminally tagged protein.

### Transfection of cells and RNAi

Procyclic-form (29-13) and bloodstream-form (90-13) *T. brucei *cells were transfected with linearized plasmid as described previously [24]. Cells were selected with either 2.5 μg/ml phleomycin for pZJM transfectants or 10 μg/ml blasticidin for pC-PTP-BLA transfected cells.

Stable cell lines carrying the pZJM vectors were cloned by plating on an agarose plate, followed by outgrowth of individual colonies, as described previously [26]. RNAi was induced by tetracycline to a final concentration of 1.0 μg/ml and cell growth monitored daily using a hemocytometer with a microscope. Semi-quantitative RT-PCR was performed as described previously [28]. Briefly, RNA samples were extracted from *T. brucei *cells using Trizol (Invitrogen) and amplified using the One-Step Taq RT-PCR system (Invitrogen). Samples of the PCR reactions were analyzed after each cycle from #20 to #24 by gel electrophoresis. For quantitative real time RT-PCR analysis, samples of total RNA were treated with Express DNase I (Ambion) at 37°C for 30 min to remove traces of genomic DNA, then heated to 75°C for 15 minutes. RNA was then converted to cDNA using Superscript III (Invitrogen) with an oligo d(T) as primer. New primers for CRK9 and α-tubulin were designed to amplify a 100 bp region in each gene. Duplicate reactions were amplified using the SYBR green master mix kit (Roche) in transparent 0.2 ml strip tubes (USA Scientific) and loaded into an MX3005P qPCR thermal cycler (Stratagene). Samples were amplified through 40 cycles and fluorescence of the SYBR green dye was used to monitor the amount of double-stranded DNA. Ct or threshold values were determined automatically using the thermal cycler software and used to calculate differences in Ct values between un-induced and induced RNAi samples. Ct differences were converted into fold differences by raising 2 to the power of the Ct difference. Fold differences were converted to percentages of mRNA levels by multiplying the reciprocals of fold differences by 100%. To verify that the fluorescence was not due to non-specific amplification, a dissociation curve was performed at the end of the 40 cycle PCR. Specific amplification was defined by the appearance of a single peak.

### Flow cytometry and karyotype analysis

To analyze the cellular DNA content, cells were harvested, washed with PBS and fixed in 70% ethanol, 10% glycerol overnight at 4°C. They were then rinsed with PBS and stained with propidium iodide (PI) as described previously [24]. Approximately 25,000 stained cells were analyzed by flow cytometry using a FacsCalibur machine (BD biosciences). The resulting histograms were further analyzed using the ModFit software (ModFit) to determine proportions of cells in G1, S and G2/M phase.

The stained cells were also examined visually using a fluorescence microscope (Olympus). Cells were categorized into 1N1K, 1N2K or 2N2K by the numbers of nuclei and kinetoplasts counted in each cell (N, nuclei; K, kinetoplasts).

### GST pull down assay

Full length genes were cloned into the pGEX6P3 vector and the resulting plasmid transferred into *E. coli *BL21(DE3) cells, which were grown to an optical density (600 nm) of 0.3. Gene expression was induced with 0.1 mM isopropyl-β-thiogalactoside (IPTG) at 30°C for three hours. Cells were harvested and suspended in lysis buffer (50 mM Tris-Cl pH 7.5, 0.3 M NaCl, 0.1% Tween-20), supplemented with a complete cocktail of protease inhibitors (Roche), and lysed by sonication at 4°C (Fisher Scientific) with 8 to 10 30-second pulses and 1-minute intervals in between. Cell debris was removed by centrifugation at 12,000 × g for 10 min and the cleared lysate loaded onto a glutathione Sepharose 4B column (GE Healthcare). Unbound proteins were removed by washing with lysis buffer and the bound proteins were eluted with 50 mM Tris-Cl pH 8 and 10 mM reduced glutathione. The yield was generally 50-100 μg of purified protein per liter of culture. The purified protein was incubated with *in vitro *transcribed/translated HA-tagged cyclins, loaded onto glutathione Sepharose 4B beads and washed three times with lysis buffer. The beads were then boiled in sample buffer and the freed proteins fractionated in SDS-PAGE followed by Coomassie blue staining. Anti-HA Western blotting was performed with the HA-7 anti-HA antibody (Sigma) at a 1:5,000 dilution followed by staining with secondary HRP-linked anti-mouse antibody (Promega) at a 1:10,000 dilution. Bands were then visualized using ECL (GE Healthcare), and exposed to X-ray film (LabSource).

### Immunofluorescence assays

For immunofluorescence experiments, cells were adhered onto poly-L-lysine (Sigma) coated cover slips, fixed with 4% paraformaldehyde at 4°C for 20 min and permeabilized with PBS and 0.2% Triton-X100 for 5 min. The cover slips were blocked for 1 hour at room temperature with PBS and 5% BSA, incubated with primary antibody for 1 hour, washed three times with PBS, and then incubated with a FITC-conjugated secondary antibody. The cover slips were washed in PBS three more times and mounted in mounting media plus DAPI (Vectashield laboratories) onto slides and sealed with wax. Slides were then examined using an inverted fluorescence microscope (Olympus). Image analysis and merging was performed using the image J software (NIH). The primary antibodies used included HA7 mouse monoclonal anti-HA antibody (1:1,000 dilution) and rat YL1/2 antibody for the basal bodies in *T. brucei *(Chemicon) (1:500 dilution). Secondary antibodies used were: FITC-conjugated anti-mouse IgG antibody (Sigma) and FITC-conjugated anti-Rat antibody (Chemicon).

## Results

### RNAi mediated ablation of cyclin and CRK homologues in procyclic form T. brucei

**Figure 1:**
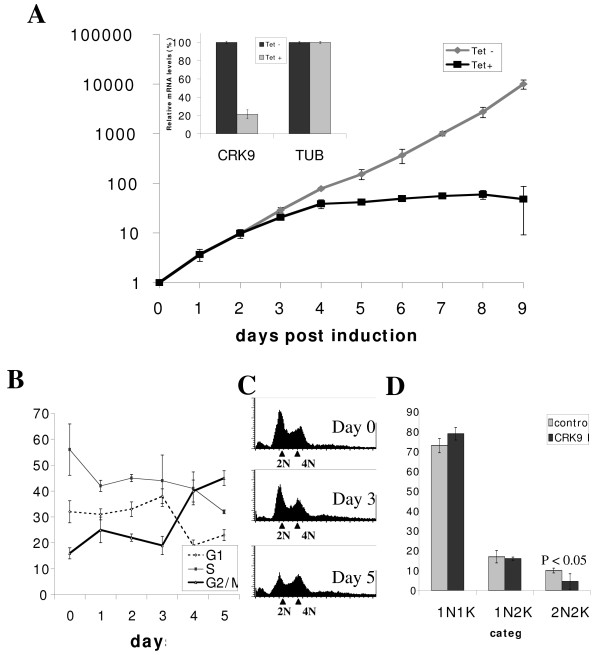
**RNAi of CRK9**. Procyclic form (29-13) cells were transfected with a pZJM vector carrying a short (300 bp) fragment of CRK9. Transfected cell lines were selected using phleomycin and cloned. **(A) **RNAi was induced by addition of tetracycline (1.0 μg/ml) and cell growth monitored daily using a hemocytometer. Daily dilution of cell cultures with fresh medium was performed when the cells grew at a linear rate. Insets show mRNA levels of CRK9 and tubulin determined by real time quantitative RT-PCR; levels are shown relative to those of un-induced cells. **(B) **Cells were harvested daily, fixed and stained with PI, followed by DNA content analysis using flow cytometry. The proportion of cells in each phase of the cell cycle was determined using the ModFit software and the proportions of G1, S and G2/M phase cells were plotted each day. **(C) **Cell sorting histograms of cell distribution by DNA contents. The small peaks next to the y-axis represent cell debris. **(D) **The PI-stained cells were also examined visually using fluorescence microscopy and the numbers of 1N1K, 1N2K and 2N2K cells determined as percentages of a total population of ~200 cells after 5 days of CRK9 RNAi (N, nucleus; K, kinetoplast).

The expression of newly identified homologues of cyclins (CYC9-10 accession Tb08.11J15.340, and Tb08.11J15.300) and CRKs (CRK7-12, accession Tb07.43M14.340, Tb11.02.5010, Tb927.2.4510, Tb03.48K5.160, Tb06.5F5.880, and Tb11.01.4130, respectively) [1,2] were each silenced in RNAi experiments for potential effect on cell cycle progression in procyclic-form *T. brucei*. Significant reduction in each of the mRNA levels was verified with semi-quantitative RT-PCR 2 days after induction of RNAi (Additional File 1, insets), and cell growth was monitored daily during the knockdown experiments and shown to be unaffected in most cases (Additional File 1) except for the CRK9 knockdown, which resulted in total growth arrest after four days (Additional File 1). The same RNAi experiment on CRK9 was repeated and resulted in the same outcome (Figure 1A). The level of CRK9 mRNA before and after 2 days of RNAi were further examined by real time quantitative RT-PCR. The result showed a significant reduction of CRK9 mRNA down to 20% of the original level after a two-day RNAi (Figure 1A inset). We thus concluded that CRK9 could be essential for procyclic cell cycle regulation whereas the other novel cyclin and CRK homologues may not be. Subsequent investigation had since been focused on CRK9.

### CRK9 depletion enriches the procyclic-form cells in G2/M phase

Timed cell samples depleted of CRK9 were stained with PI and analyzed in flow cytometry. During the 5 days of RNAi, the proportion of cells in G2/M phase (4C) increased gradually from 17% to 44% whereas the G1 cells (2C) decreased from 55% to 31% and the S-phase cells reduced from 32% to 22%. (Figure 1B). Cells without RNAi did not appreciably change the distribution of their DNA content over the same course of experiment (data not shown). This result suggests that cells depleted of CRK9 are enriched in G2/M phase.

To find out if kinetoplast replication still proceeded in the RNAi cells arrested in G2/M phase, the PI-stained cells were examined with a fluorescence microscope for the numbers of nuclei (N) and kinetoplasts (K) in each cell. The kinetoplast is known to begin replicating at about the same time as nuclear DNA but the kinetoplast segregation is completed prior to the onset of mitosis. Thus, cells stopped prior to mitosis are known to become enriched in 1N2K cells and generate large numbers of anucleate progenies (zoids) due to the apparent capability of replicated kinetoplast/basal body in initiating cytokinesis without mitotic completion in the procyclic form [16,21]. Those stopped after completion of mitosis but prior to cytokinesis initiation are enriched with 2N2K and XNXK cells [24,27,32,33]. The CRK9 depleted cells did not indicate any detectable increase in 1N2K, 2N2K or XNXK cells, but, instead, showed a slight increase (from 72% to 80%) in 1N1K cells and a corresponding decrease (10% to 3%) in 2N2K cells (Figure 1C) without any zoid formation. Together, the results suggest an inhibited mitosis and a blocked kinetoplast/basal body replication/segregation with an anticipated inhibition of cytokinesis. These multiple blocks have been supported by the observed enrichment of CRK9 knockdown cells in G2/M phase (see Figure 1B). The 8% increase in 1N1K cells could be provided by the 2N2K cells already present before RNAi induction. They dropped by 7% thereafter, suggesting a normal proceeding of cytokinesis among them. CRK9 thus may not play a direct role in controlling the progression of cytokinesis but could regulate its initiation by controlling the kinetoplast/basal body replication/segregation. The apparent G2/M arrest in CRK9-depleted cells could be an indirect consequence from an inhibited cell division from a blocked kinetoplast/basal body replication/segregation. These probabilities will be further addressed in the experiments described below.

### Depletion of CRK9 causes a drastic morphological change

**Figure 2:**
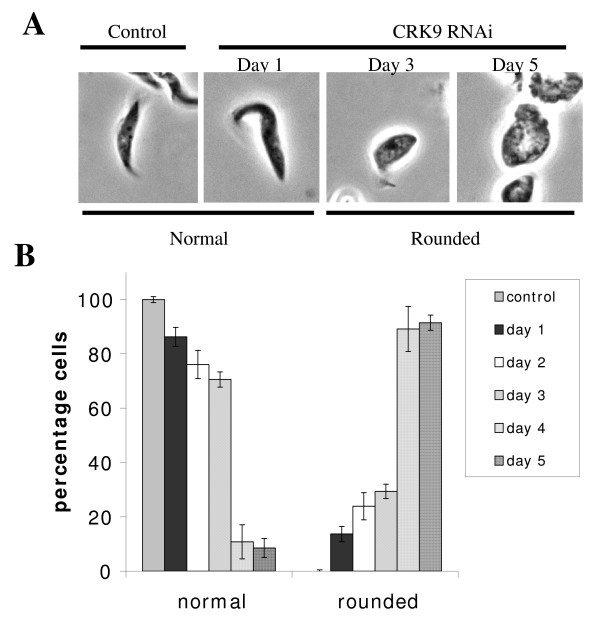
**Morphology of CRK9 depleted cells**. The control cells (without RNAi induction) and the cells depleted of CRK9 by RNAi from the same set of experiments in Figure 1 were fixed and examined by light microscopy 1-5 days after induction of RNAi (as indicated in the top panel, **2A**). The cells were classified as normal when the ratio of lengths between the major and minor axis was greater than 5:1but as morphologically rounded when the ratio was below 3:1. The frequency of cells appearing in normal and rounded morphology was tabulated from ~200 cells in each sample (bottom panel, **2B**).

An additional observation made on the CRK9-depleted cells was that, after RNAi induction, the cells gradually lost the original morphology and turned into a round shape (Figure 2A). The normal and rounded cells were defined by the ratio between the major and the minor axis lengths. For ratios higher than 5:1, the cells were defined as normal, whereas for ratios less than 3:1, the cells were defined as rounded. The change took place slowly with 20% of the round-shaped cells appearing on day 2, 24% on day 3, 87% on day 4 and 91% on day 5 (Figure 2B), whereas 97% of the control cells retained the normal morphology throughout the entire time course. When the cells were stained with L8C4 antibody against the paraflagellar rod protein in flagellum, the flagellum and the associated basal body were attached to one side of the cell as in the wild type, even though the shape of the cells has changed drastically (Additional File 2). The morphological change has thus apparently not caused a defective flagellar biogenesis. The procyclic form cells are known to undergo an active microtubule corset extension toward the posterior end during the G1 to S-phase transition that does not occur in the bloodstream form (Tu and Wang, 2005). It is thus possible that a normal progression of microtubule extension during G1/S transition coupled with a blocked kinetoplast/basal body replication/segregation in the CRK9 depleted cells could have caused the morphological change (see below).

### Kinetoplast and basal body segregation is impaired in CRK9 depleted cells

**Figure 3:**
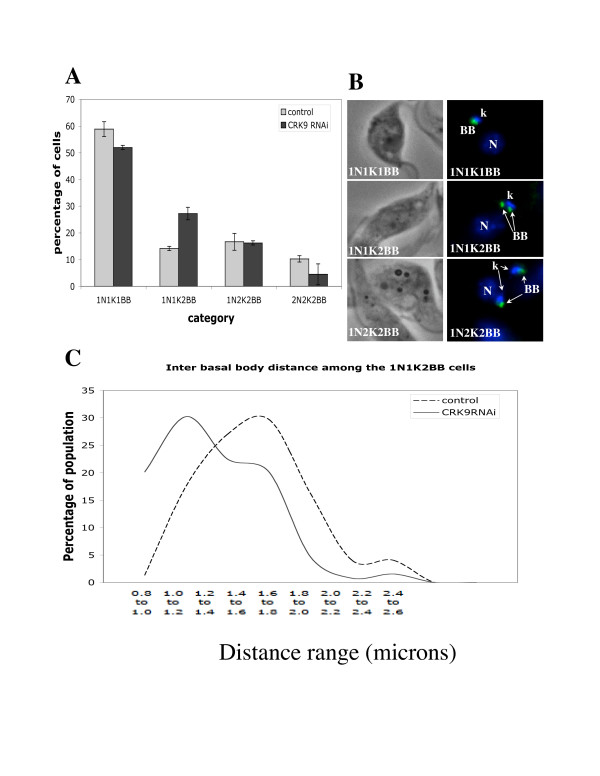
**Analysis of basal body replication and segregation in CRK9 depleted cells**. Cells induced for CRK9 RNAi for 5 days were fixed and stained with YL1/2 antibody to show the basal bodies. DAPI was used to stain the nuclei and kinetoplasts. Cells were classified as 1N1K1BB (N, nuclei, K, kinetoplast, BB, basal body), 1N1K2BB, 1N2K2BB or 2N2K2BB cells and tabulated from a population of ~200 cells in each sample (**A**). Phase and DAPI-stained images of three different types of cells, 1N1K1BB, 1N1K2BB and 1N2K2BB are shown in panel (**B**). The distances between the two basal bodies in ~200 1N1K2BB cells were measured using the ImageJ software and compared between the control and CRK9 depleted cells (**C**).

A specific segregation between the two basal body/kinetoplast pairs is essential for initiating cytokinesis in procyclic *T. brucei *[34,35]. It involves extension of the corset of microtubules and migration of the newly formed basal body/kinetoplast pair toward the posterior end of the cell [16]. We hypothesized that if a CRK9 knockdown would result in a retarded segregation between the two basal body/kinetoplast pairs while the microtubule extension proceeds normally from the basal bodies, the microtubule organizing center, it could block cytokinesis and cause distorted cell morphology as well. To test this hypothesis, we further classified the CRK9 depleted cells into 1N1K1BB (one nucleus, one kinetoplast, one basal body), 1N1K2BB, 1N2K2BB, and 2N2K2BB cells (examples of which are shown in Figure 3B) with a pair of still joined basal bodies counted as one basal body. With the tabulation conducted in this manner, we found no evidence of any 1N2K1BB or 2N2K1BB cells, agreeing with the previous observation that kinetoplast segregation depends on basal body segregation (Robinson et al., 1995). It also suggests that a failed segregation between two basal body/kinetoplast pairs could be caused by an inhibited kinetoplast replication/segregation while the two replicated basal bodies are trying to separate.

Strikingly, the CRK9 depleted cells, after 4 days of RNAi induction, showed an increase from 14% to 28% in 1N1K2BB cells (Figure 3, panel A). At the same time, however, the 1N1K1BB and 2N2K2BB cells decreased by 8% and 7%, respectively, while 1N2K2BB cells remained relatively constant. A simple explanation for this population shift could be that 1N1K1BB cells were converted to 1N1K2BB whereas 2N2K2BB were divided to produce 1N1K1BB, which then proceeded to 1N1K2BB without being further converted to 1N2K2BB or 2N2K2BB while CRK9 was depleted. This scheme suggests no defect in converting 1N1K1BB to 1N1K2BB (thus no difficulty in basal body replication/segregation), but difficulty in further changing 1N1K2BB to 1N2K2BB and beyond. It is thus likely that while basal body replication appears undisturbed, nuclear division and kinetoplast replication/segregation could be blocked by a CRK9 knockdown, which may lead indirectly to blocked segregation between two replicated basal bodies.

To investigate if this interpretation could be correct, we measured the distance between the two basal bodies in 1N1K2BB cells from the control and the 5-day CRK9 RNAi cells. There was a significant decrease in the mean distance from 1.36 μm (standard deviation ± 0.08) in the control to 1.08 μm (standard deviation ± 0.12) in the CRK9-depleted 1N1K2BB cells (Figure 3C). It is thus possible that this retarded segregation between the two basal bodies, caused probably by the failure in kinetoplast replication/segregation, could lead to inhibition of cytokinetic initiation and morphological distortion. An examination of the distances between two basal bodies in 1N2K2BB and 2N2K2BB cells showed no apparent difference from the control (data not shown). Thus, apparently, when the two kinetoplasts have already been formed, basal body segregation proceeds normally. CRK9 depletion thus enriches the cells, in which kinetoplast replication/segregation is affected.

### CRK9 was found to interact with cyclin B2 in vitro

The apparent inhibition of kinetoplast replication/segregation in CRK9-ablated cells raises the question on whether CRK9 acts also as a genuine cyclin-dependent kinase in procyclic-form *T. brucei*. Comparing with the other CRKs from *T. brucei *(Additional File 3), CRK9 has several extra insertions upstream, downstream and within the Ser/Thr kinase domain and a PSSGLLR motif instead of the well-conserved PSTAIRE motif required for cyclin binding. A structural modeling of CRK9 with the EasyPred 3D server generated a predicted structure most closely matching that of *T. brucei *CRK1 (Additional File 4, top panels). The invariant Lys, the Asp-Phe-Gly motif and the Gly rich box all locate to the predicted active site and their positions are super-imposable with those from the predicted structure of CRK1, the kinase playing an essential role in regulating G1/S passage in *T. brucei*. The PSSGLLR motif in CRK9 is predicted to be unstructured (Additional File 4, bottom panel), whereas the PSTAIRE motifs are known to generally assume a helical structure [36]. Since CRK1 is known to bind the G1 cyclin E1 in controlling G1/S passage and the mitotic cyclin B2 in regulating G2/M transition in *T. brucei *[24,26,28], a possible involvement of CRK9 in binding to cyclin E1 or cyclin B2 and a role in nuclear cycle regulation cannot be ruled out in view of its predicted structure.

**Figure 4:**
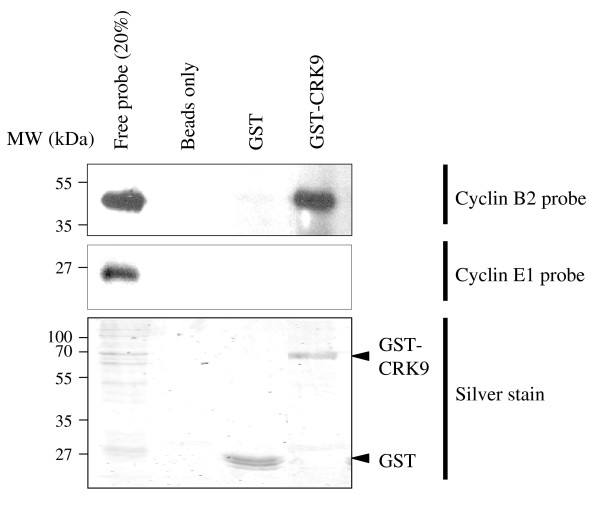
**CRK9 interacts with cyclin B2**. Recombinant GST-CRK9 was purified from transformed *E. coli *and incubated with the *in vitro *transcribed/translated HA-tagged cyclins E1 and B2, respectively. The GST protein complexes were bound to glutathione agarose, washed extensively, and analyzed by SDS PAGE followed by anti-HA Western blotting (top and central panels, as indicated) or total protein staining as loading controls (bottom panel). GST and glutathione beads without fused proteins attached served as negative controls.

GST-CRK9 was expressed in *E. coli*, purified and tested for its bindings to cyclin E1 and cyclin B2, the only two functional cyclins in *T. brucei*, in an *in vitro *pull down assay. Neither of the two cyclins bound either glutathione beads or GST (Figure 4, beads only and GST lanes). GST-CRK9 did not have an apparent interaction with cyclin E1 (Figure 4, middle row) as less than 0.1% of the amount of cyclin E1 input could be found associated with GST-CRK9. However, GST-CRK9 did bind to cyclin B2 as 21% of the cyclin B2 input was recovered from the bound GST-CRK9 (Figure 4, top row). This apparent binding between CRK9 and the mitotic cyclin B2 agrees well with the enrichment of G2/M cells when CRK9 is depleted. CRK9 could be thus a cyclin B2-dependent kinase involved in regulating G2/M passage in the procyclic cells.

### Localization of CRK9

**Figure 5:**
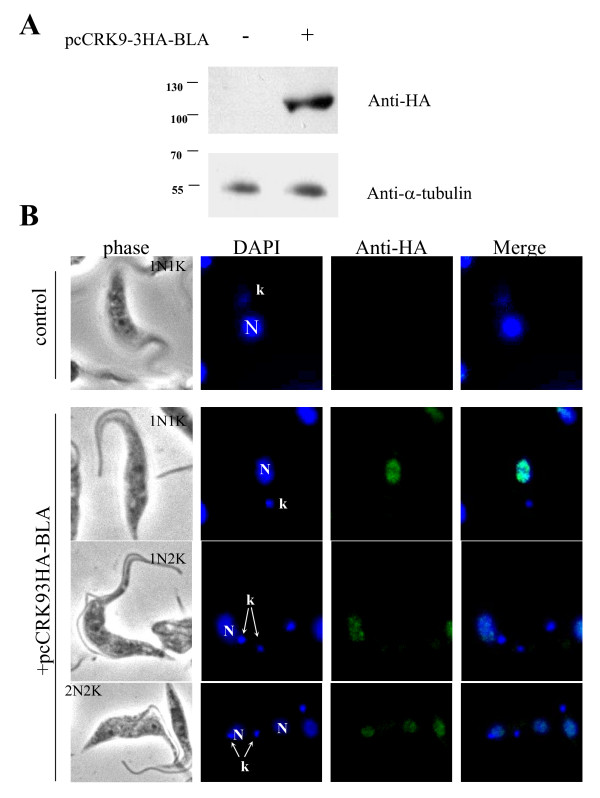
**Localization of CRK9 in procyclic cells**. CRK9 fused to the 3xHA tag was inserted into the pc-BLA-PTP vector. After linearization, transfection into *T. brucei*, and selection using blasticidin (10 μg/ml), cells expressing CRK9-3HA at the endogenous level were obtained. Transfected and control cells were analyzed by Western blotting for the HA tag as indicated (**A**). The blot was also probed with anti α-tubulin to verify equal sample loading. Localization of CRK9 was determined by fixing the transfected cells and staining them with a FITC-conjugated anti-HA antibody (**B**). DAPI was used to visualize the nucleus and kinetoplast and merged images were created using the imageJ software.

CRK9 tagged with 3xHA at the C-terminus was expressed in procyclic cells to the putative endogenous level by replacing one allele of the encoding gene with a linearized construct of 3xHA tagged CRK9 through homologous recombination. Western blot analysis of the transfected cell lysate using an anti-HA antibody showed that a single band at approximately 105 kDa, corresponding to the predicted molecular weight of CRK9, was identified (Figure 5A). The same blot was stained with anti-α-tubulin antibody as a sampling control (Figure 5A). Similar experiments were performed on the bloodstream form of *T. brucei*. The result from Western blot showed also a 105 kDa protein by anti-HA antibody (Additional File 5A).

Using the same anti-HA antibody to determine the localization of CRK9-3HA by immunofluorescence, the endogenously expressed CRK9-3HA was found to localize in the nucleus in 1N1K, 1N2K and 2N2K cells (Figure 5B). In a similar immunofluorescence assay of the bloodstream form 1N1K and 1N2K cells, the endogenously expressed CRK9-3HA was also localized in the nucleus (Additional File 6). However, an extensive examination of CRK9 localization throughout the entire cell cycle has not yet been conducted to rule out the possibility that CRK9 might trans-localize out of the nucleus in late mitotic phase to participate in cytokinetic initiation as does the Aurora B kinase in *T. brucei *[37].

### Depletion of CRK9 has no apparent effect on the growth of bloodstream form cells

The retarded basal body/kinetoplast segregation caused by CRK9 depletion in the procyclic-form cells could constitute the mechanism of inhibited cell division [16,34]. But since a similar kinetoplast/basal body segregation does not occur in the bloodstream form [38] a similar inhibitory effect of cytokinetic initiation may not happen in the bloodstream form when CRK9 is ablated. The bloodstream-form cells (90-13) were transfected with the pZJM-CRK9 plasmid, selected with phleomycin, and cloned. RNAi induced by tetracycline for 2 days resulted in an effective reduction of the mRNA for CRK9 down to 30% of the original level when monitored by real time quantitative RT-PCR (Additional File 5, inset). This depletion of CRK9 did not, however, result in a detectable effect on cell growth, as the cells induced for RNAi grew at about the same rate as the un-induced control (Additional File 5). This missing inhibitory effect from CRK9 depletion in the bloodstream form could be attributed to an apparent lack of required kinetoplast/basal body segregation for cytokinetic initiation. The inhibited mitosis from depleted CRK9 in procyclic form could be attributed to potentially distinctive mechanisms controlling mitosis in the two forms (see Discussion), or, the blocked cytokinesis in procyclic form may exert an inhibitory effect on mitosis. Alternatively, the possibility that the 30% of CRK9 remaining after RNAi induction in the bloodstream cells could be sufficient in maintaining the cells in the wild type phenotype has not yet been totally ruled out at present.

## Discussion

In the present study, we concluded a preliminary functional analysis of all the structural homologues of cyclin and CRK identified in *T. brucei *[1,2]. From the previous investigations, CycE1/CRK1 is known to regulate the G1/S passage [24-26,28], CycE1/CRK2 controls the posterior extension in the procyclic form during G1/S transition [28,39], whereas CycE1/CRK3 and CycB2/CRK3 are involved in regulating the passage from the G2 phase to the mitotic phase [24,26-28]. The new experimental data from the current study covered the rest of the homologues that have not yet been analyzed. They added one more protein kinase CRK9 that plays an essential role in promoting the growth of procyclic form but apparently not the bloodstream form of trypanosome. For the rest of the newly as well as the previously identified homologues of cyclins and CRKs, their knockdowns showed little apparent effect on trypanosome cell growth. Among those CRK's whose knockdowns in the present study did not register any phenotype, their sequences indicate that they either lack or have mutated certain residues in the glycine-rich box or the conserved glutamate residue immediately downstream of the gatekeeper that are known to be a part of the kinase active site (see Additional File 3). They could be thus either pseudogenes or their protein products may not possess any kinase activity. If they still play any role at all in cell cycle regulation, they might perform either an auxiliary or redundant function.

In other eukaryotic organisms, certain cyclin/Cdk pairs are dispensable, because they are known to play an auxiliary role. For example, cyclin D/Cdk4 in mammalian cells promotes exit from G0 into G1 by countering the activity of anti-proliferating agent such as Rb and p21 [40-42]. These auxiliary cyclin/Cdk pairs are generally required for stress responses and adaptive conditions [6,43-45]. Thus, CycE2, E3, E4, B1, B3 and 10-11, CRK4, 6-8,10-12, found without an apparent function in cell growth in the previous and present studies, could be the auxiliary proteins dispensable for cell cycle regulation in *T. brucei*. Given that the latest bioinformatics analysis of the *T. brucei *kinome appears complete, this work may have completed the identification of all the primary cell cycle regulators in trypanosome.

Further analysis of CRK9 indicated that the procyclic form is enriched in the G2/M phase upon the depletion of CRK9. The protein is localized to the nucleus and binds to CycB2 *in vitro*. These indications suggest that CRK9 may form complex with CycB2 and play an essential role in regulating the G2/M passage in procyclic-form *T. brucei*.

The arrested mitosis in procyclic cells depleted of CRK9, however, does not lead to appreciable formation of anucleated cells as observed previously from depleting the mitotic kinase CRK3 [26]. This is attributed to the fact that CRK9 plays also another role in regulating cytokinesis by controlling kinetoplast replication/segregation in the procyclic trypanosome. Thus, in addition to a mitotic arrest, CRK9 ablation leads also to a failure in kinetoplast replication/segregation, which causes a block of basal body segregation and cytokinetic initiation in the procyclic form. This blockade is apparently also responsible for a drastic morphological change of cells to a round shape, which has not yet been observed previously when cytokinesis was inhibited in the procyclic form of *T. brucei *by other means [32,33,35,46]. Thus, CRK9 may be involved in cytokinetic regulation with a mechanism completely different from that of the phosphatases PP1 or PP2A, targets of okadeic acid inhibition, (Das et al., 1994), TbAUK1 [32,46] and TbPLK [33]. These enzymes are known to control cytokinesis, but their depletion does not cause a morphological change of the procyclic cells. Okadaic acid, the inhibitor of PP1 and PP2A, is known to arrest the cytokinesis of procyclic cells resulting in a single kinetoplast, a single basal body, a single flagellum but multiple nuclei in each cell. Thus, cytokinesis is blocked at a very early phase prior to kinetoplast/basal body replication by this inhibitor. A depletion of TbAUK1, however, results in cytokinesis-arrested procyclic cells with two widely segregated kinetoplast/basal body pairs, indicating that TbAUK1 acts downstream from CRK9. A TbPLK depletion leads to cytokinesis-arrested cells with multiple nuclei, kinetoplasts, basal bodies, flagella, and is known to play a role downstream from that of TbAUK1 in regulating cytokinesis [33,46]. Thus in a series of regulators of cytokinetic initiation, their order of actions is most likely; the phospatases→CRK9→TbAUK1→TbPLK. Furthermore, an active extension of microtubule corset toward the posterior end is known to take place only during the G1/S transition in the procyclic form [16,28,39,47]. It was postulated that this extension is coupled with the segregation between the two kinetoplast/basal body pairs. Assuming that the microtubule extension might continue while the kinetoplast segregation is inhibited by a depletion of CRK9, a distortion of cellular morphology could result from such a lack of coordination. As for the question on how a nuclear protein CRK9 plays a role in controlling basal body/kinetoplast replication/segregation, we don't have a ready answer yet. But we could hypothesize that CRK9 is a part of a signaling network coordinating basal body replication/segregation with nuclear duplication. It may phosphorylate a yet un-identified protein(s) that promotes basal body replication/segregation in procyclic cells. A pursuit of the mechanism of CRK9 regulation of cytokinesis will be thus a major interest in our future investigations.

CRK9 thus distinguishes itself from the other CRKs in *T. brucei *by playing essential roles in controlling both mitosis and cytokinesis in the procyclic trypanosome. There is only one other protein kinase in *T. brucei *known to play essential roles in both mitosis and cytokinesis. It is an orthologue of Aurora B, TbAUK1, which does not belong to the CRK family [32,46]. Considerable knowledge has been accumulated on this protein kinase in recent years. It is a component of the unique chromosomal passenger complex that is confined in the nucleus during mitosis, but migrates out of the nucleus and trans-localize to the potential cleavage furrow on the dorsal side of the cell in telophase to initiate cytokinesis [37]. CRK9 could have a similar pattern of trans-localization like that of TbAUK1 toward the end of mitosis, i.e., to move out of the nucleus and become associated with the organelles involved with cytokinetic initiation. Only a detailed further analysis of CRK9 localization in a synchronized cell population would shed some light on this interesting possibility.

A depletion of TbAUK1 leads to an elimination of the spindle structure in the nucleus, mitotic arrest and cytokinetic block with two kinetoplast/basal body pairs in the procyclic cells, which are similar, albeit not identical, to that observed from CRK9 depletion. The multiple functions of TbAUK1 can be, however, observed in both procyclic and bloodstream forms of trypanosome [32,46]. A depletion of TbAUK1 from the bloodstream form resulted in multiple kinetoplasts/basal bodies/flagella, reflecting a dissociation between organelle multiplication and cytokinetic initiation in bloodstream form. The lack of a role of CRK9 in controlling cytokinesis in the bloodstream form could be also attributed to such a dissociation, i.e., cytokinesis could still proceed while the kinetoplast replication/segregation has failed.

The absence of any effect on the mitosis in bloodstream form from knocking down CRK9 is a little more difficult to comprehend at the present time. But one did observe different phenotypes between the two forms when mitotic cyclin B2 or mitotic protein kinase CRK3 was knocked down in the previous studies [24,26,27]. Mitosis in the procyclic form was stopped with a nucleus approximately twice the size of that in the G1 phase. But the same knockdowns in bloodstream form resulted in an extremely large aggregate of apparently un-segregated nuclei, suggesting that, without a mitotic exit, the cells can still enter a new G1 phase repeatedly. A check point on mitosis could be thus absent from the bloodstream form though it may be still present and functioning in the procyclic form. Assuming that the role of CRK9 in regulating G2/M passage may depend on its interaction with some of the yet unidentified check point proteins, it could explain why CRK9 functions in one but not the other form.

## Conclusion

In summary, we have investigated the role of CRK9 in trypanosome cell cycle regulation, and found it functioning only in the procyclic form in controlling mitosis as well as kinetoplast replication/segregation. It is the second protein kinase in *T. brucei *next to TbAUK1 showing a dual role in controlling both mitosis and cytokinesis except that it functions only in one of the two forms. CRK9 will be thus a useful tool for future study on the coordination between mitosis and cytokinesis and the distinctive mechanisms in this coordination between procyclic and bloodstream forms.

## Authors' contributions

SG and CCW conceived and designed the study. SG carried out all experimental procedures. SG and CCW wrote the paper. All authors read and approved the final version of the manuscript.

## Supplementary Material

Additional file 1 **RNAi of the newly identified cyclin and CRK homologues**. The data shows the effect of RNAi on each individual novel cyclin and CRK. Procyclic form (29-13) cells were transfected with pZJM vectors carrying short (300-500 bp) fragments of the newly identified cyclin and CRK genes as indicated. Cell lines were selected using phleomycin and cloned. RNAi was induced by addition of tetracycline (10 μg/ml) and cell growth monitored daily using a hemocytometer. Insets show mRNA levels after RNAi for 2 days estimated by semi-quantitative RT-PCR. α-Tubulin was included as a loading control. Click here for file

Additional file 2 **Morphology of CRK9 depleted cells**. The data shows additional staining and morphology of CRK9 depleted cells to highlight the microtubules and the flagellum. Control and cells depleted of CRK9 by RNAi were fixed, stained with anti-tyrosylated tubulin (marker for basal bodies) YL1/2 and anti-paraflagellar rod antibody (L8C4) and examined by fluorescence microscopy. Click here for file

Additional file 3 **Sequence alignment among the newly identified CRK homologues**. The figure shows an alignment of the newly identified CRK homologues with the three CRKs 1, 2 and 3 whose functions have been demonstrated in previous studies, using the MacVector software. Residues important for catalysis, binding or the putative active site are indicated. A cartoon depicting the various domain structures of CRK9 compared with the well characterized CRK1-3 is shown in the top panel. Click here for file

Additional file 4 **Predicted structure of CRK9**. The figure shows the predicted structure of CRK9, obtained by submitting the sequence of CRK9 to the EasyPred 3D structure prediction server, . The predicted structure of CRK1 is shown alongside that of CRK9 (top panel). Some of the important residues are highlighted in colors (red, catalytic aspartic acid, blue, catalytic lysine, dark green, GxGxxG motif required for ATP binding, brown, PSTAIRE motif required for cyclin binding). The structures of the two proteins were also overlaid (bottom panel) and crucial residues highlighted. Click here for file

Additional file 5 **CRK9 RNAi in bloodstream form cells**. The data presented here shows that CRK9 depletion has no effect on bloodstream form cells. The pZJM-CRK9 vector was transfected into 90-13 bloodstream form *T. brucei *cells, selected with phleomycin and followed by single cell cloning. RNAi was induced as previously described and cell number monitored daily. Real time quantitative RT-PCR was used to monitor the depletion of CRK9 after 2 days of RNAi (insets); with α-tubulin serving as the loading control. Click here for file

Additional file 6 **Localization of CRK9 in bloodstream form cells**. The data shows that CRK9 localizes to the nuclei of bloodstream form cells. The pc-CRK9-3HA-BLA plasmid was linearized, transfected into 90-13 bloodstream form *T. brucei *cells, and stable cell lines were selected using blasticidin (10 μg/ml) and cloned to express CRK9-3HA at the endogenous level. Transfected and control cells were analyzed by Western blotting for the HA tag as indicated (**A**). The blot were stripped and probed with anti-α-tubulin antibody to verify equal sample loading. Localization of CRK9 was determined by fixing and staining the transfected cells with a FITC-conjugated anti-HA antibody (**B**). DAPI was used to visualize the nucleus and kinetoplast and merged images were created using the imageJ software. Click here for file
