# Supplementary figures and images for: CRK9 contributes to regulation of mitosis and cytokinesis in the procyclic form of Trypanosoma brucei
Source: BMC Cell Biol. 2009 Sep 21;10:68. doi: 10.1186/1471-2121-10-68 (PMC2754446; doi:10.1186/1471-2121-10-68)

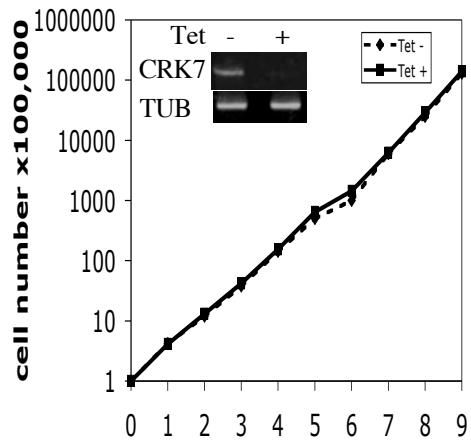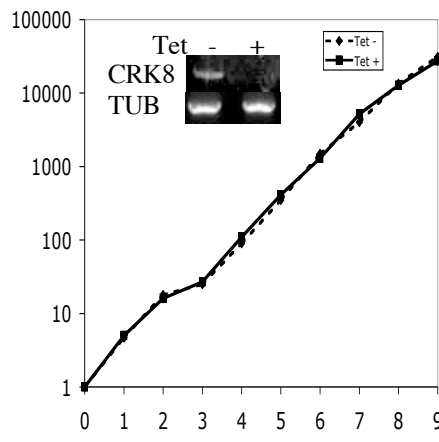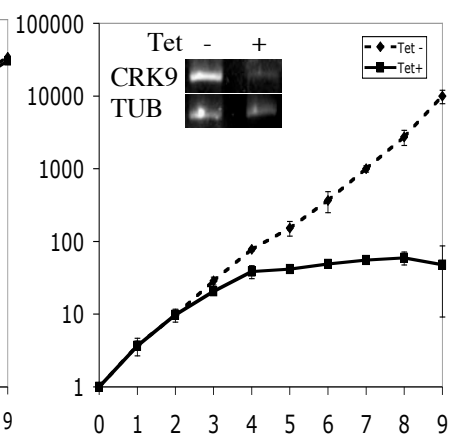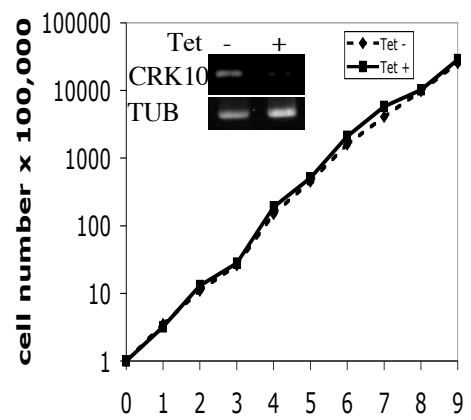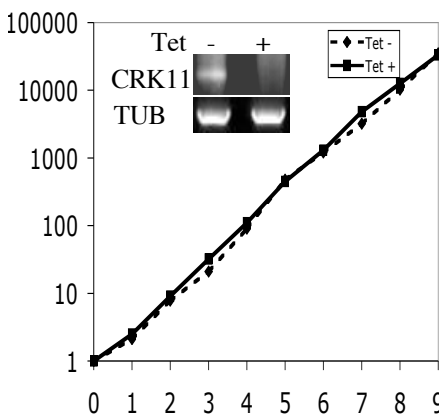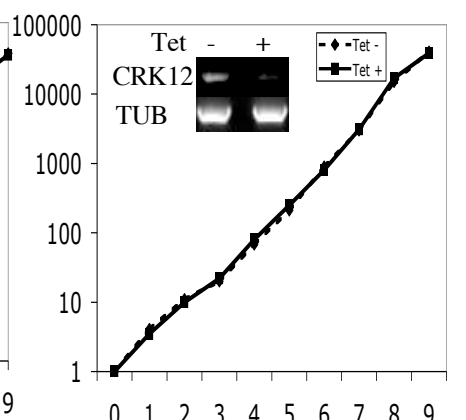

days post induction

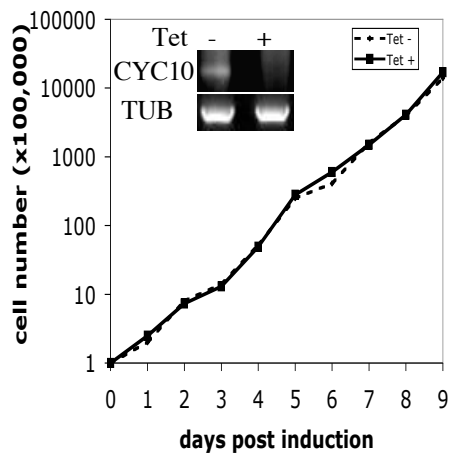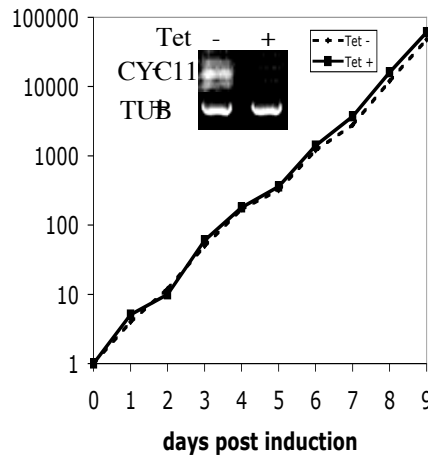

days post induction

Supplement: Additional file 1 — RNAi of the newly identified cyclin and CRK homologues. The data shows the effect of RNAi on each individual novel cyclin and CRK. Procyclic form (29-13) cells were transfected with pZJM vectors carrying short (300-500 bp) fragments of the newly identified cyclin and CRK genes as indicated. Cell lines were selected using phleomycin and cloned. RNAi was induced by addition of tetracycline (10 μg/ml) and cell growth monitored daily using a hemocytometer. Insets show mRNA levels after RNAi for 2 days estimated by semi-quantitative RT-PCR. α-Tubulin was included as a loading control. [file 1471-2121-10-68-S1.PDF]

Control

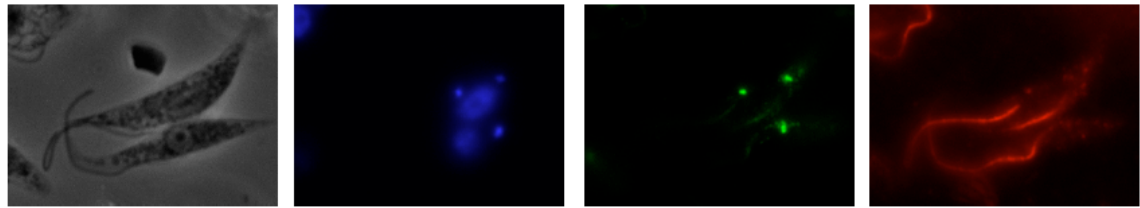

1N2K

1N1K

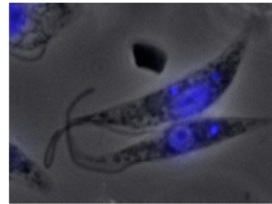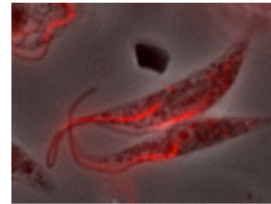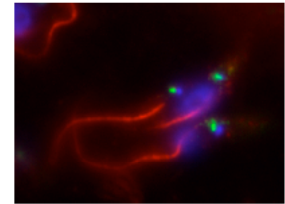

CRK9 RNAi, day 5

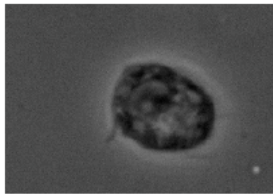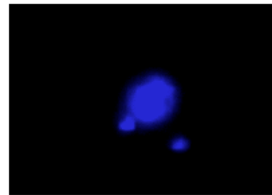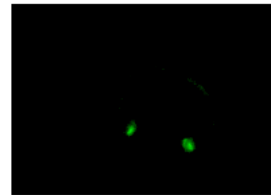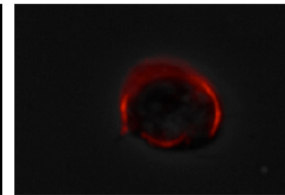

1N2K

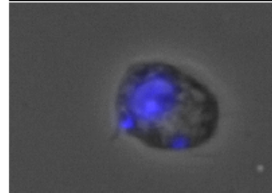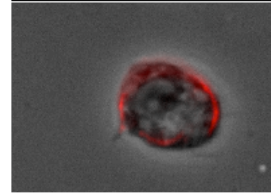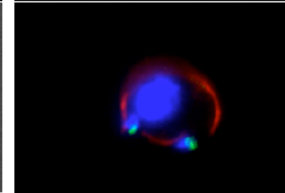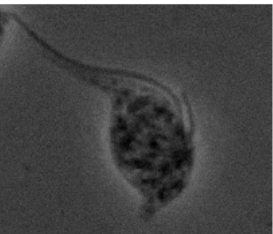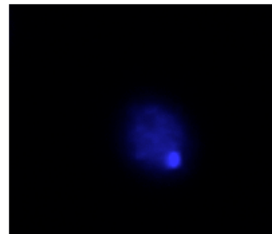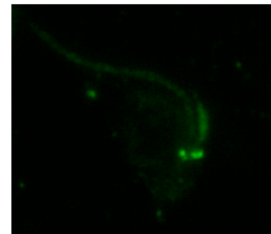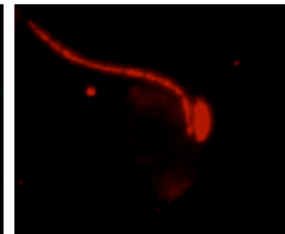

1N1K

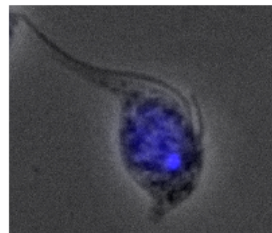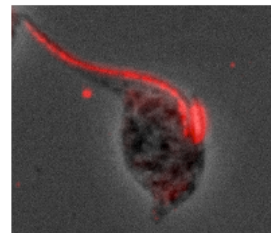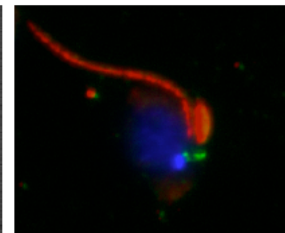

Phase

DAPI

YL1/2

L8C4

Merge,  
Phase/DAPIMerge, Phase/  
L8C4Merge,  
DAPI+YL1/2  
+L8C4

Supplement: Additional file 2 — Morphology of CRK9 depleted cells. The data shows additional staining and morphology of CRK9 depleted cells to highlight the microtubules and the flagellum. Control and cells depleted of CRK9 by RNAi were fixed, stained with anti-tyrosylated tubulin (marker for basal bodies) YL1/2 and anti-paraflagellar rod antibody (L8C4) and examined by fluorescence microscopy. [file 1471-2121-10-68-S2.PDF]

Predicted CRK1 structure

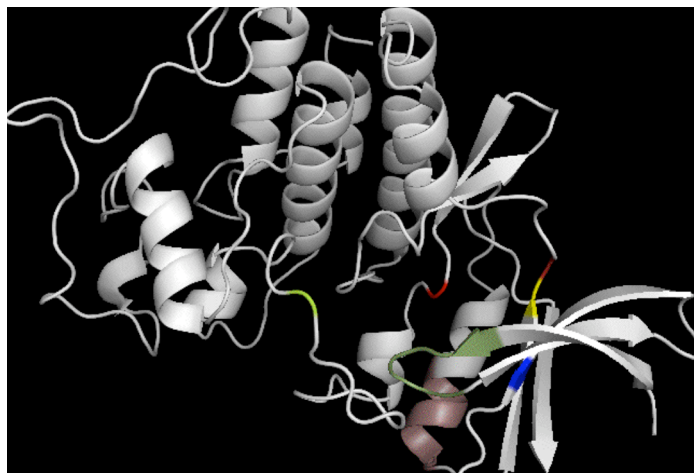

Predicted CRK9 structure

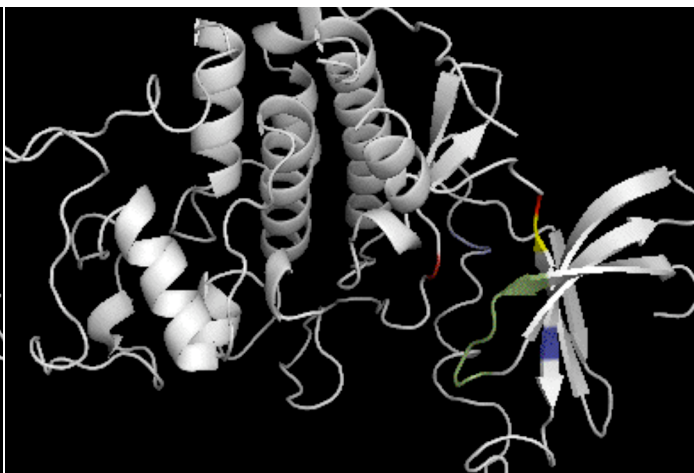

## Overlay

Entire structure

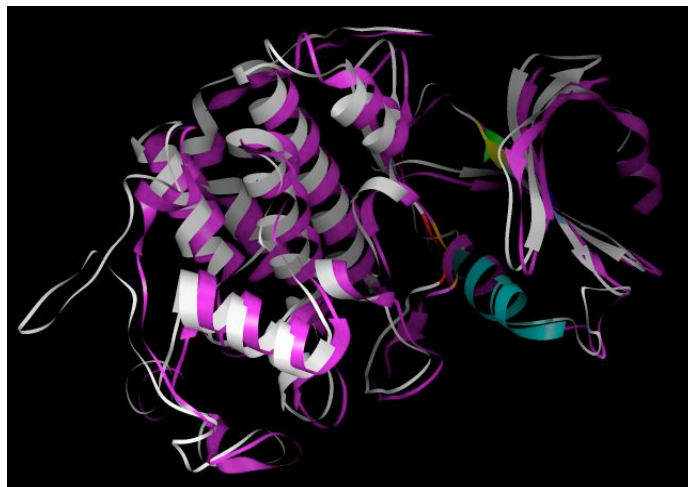

Active site

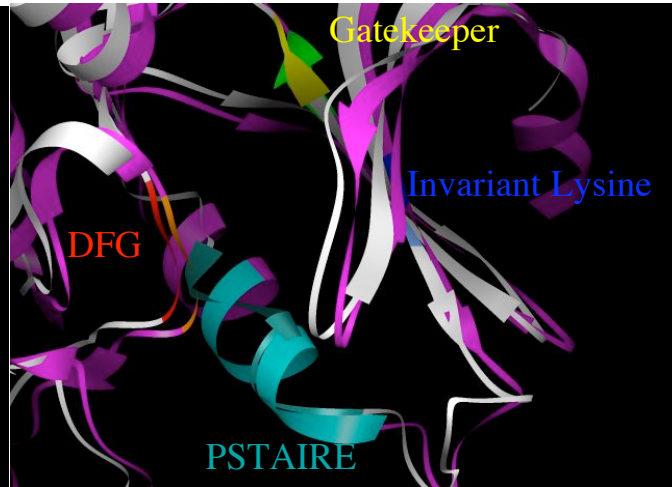

Supplement: Additional file 4 — Predicted structure of CRK9. The figure shows the predicted structure of CRK9, obtained by submitting the sequence of CRK9 to the EasyPred 3D structure prediction server, . The predicted structure of CRK1 is shown alongside that of CRK9 (top panel). Some of the important residues are highlighted in colors (red, catalytic aspartic acid, blue, catalytic lysine, dark green, GxGxxG motif required for ATP binding, brown, PSTAIRE motif required for cyclin binding). The structures of the two proteins were also overlaid (bottom panel) and crucial residues highlighted. [file 1471-2121-10-68-S4.PDF]

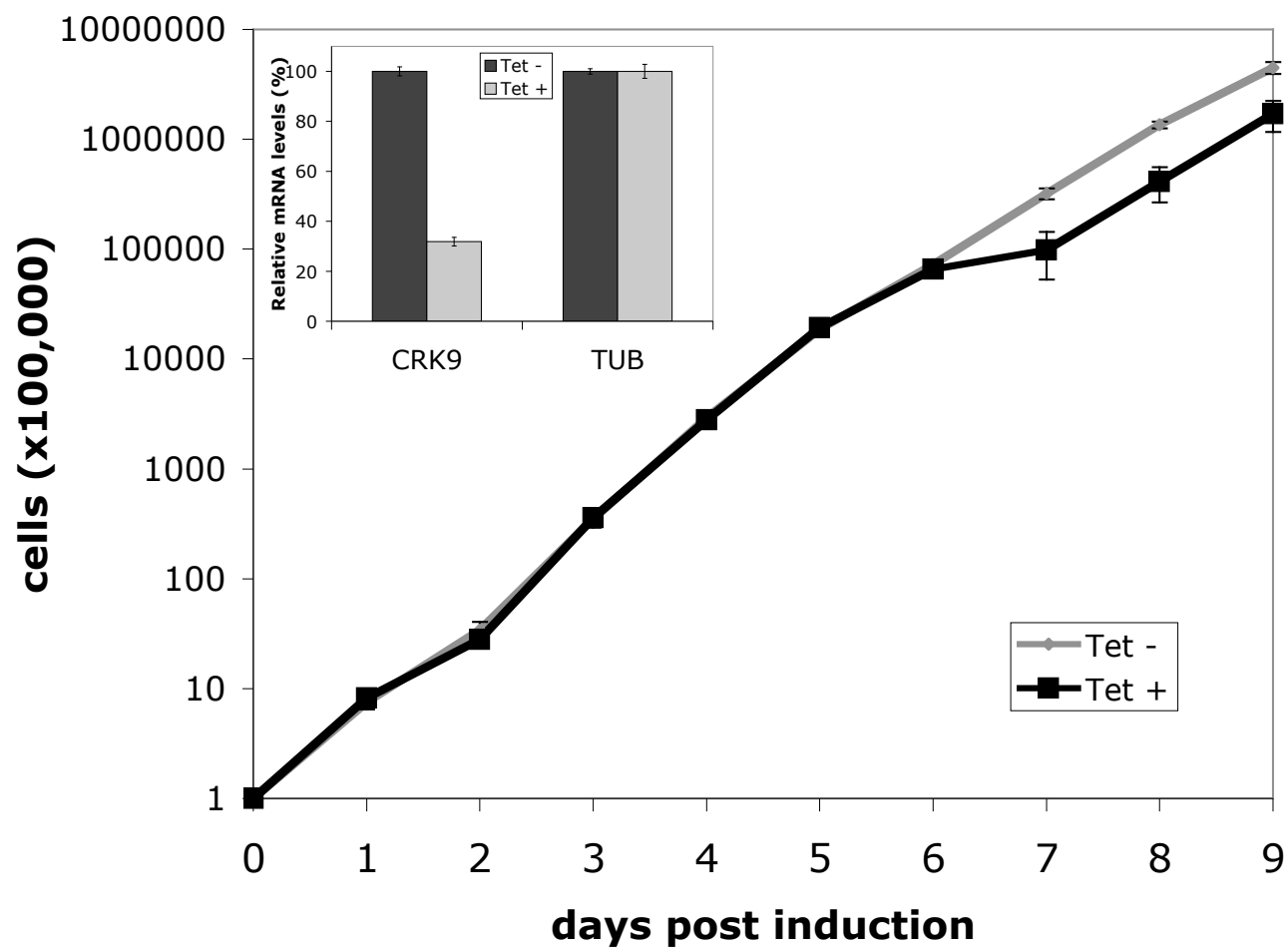

Supplement: Additional file 5 — CRK9 RNAi in bloodstream form cells. The data presented here shows that CRK9 depletion has no effect on bloodstream form cells. The pZJM-CRK9 vector was transfected into 90-13 bloodstream form T. brucei cells, selected with phleomycin and followed by single cell cloning. RNAi was induced as previously described and cell number monitored daily. Real time quantitative RT-PCR was used to monitor the depletion of CRK9 after 2 days of RNAi (insets); with α-tubulin serving as the loading control. [file 1471-2121-10-68-S5.PDF]

**A**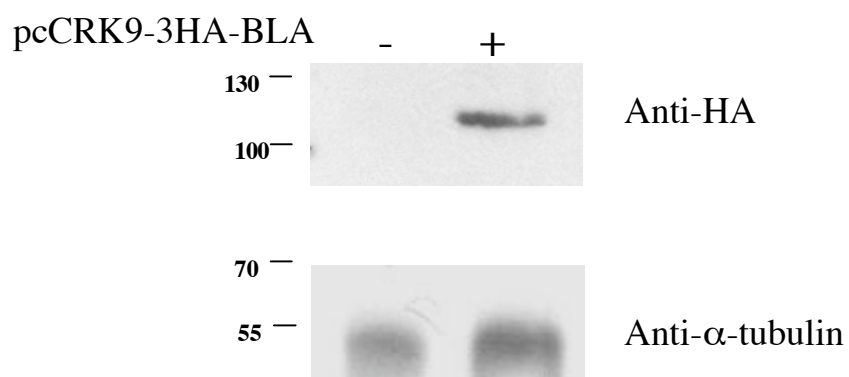**B**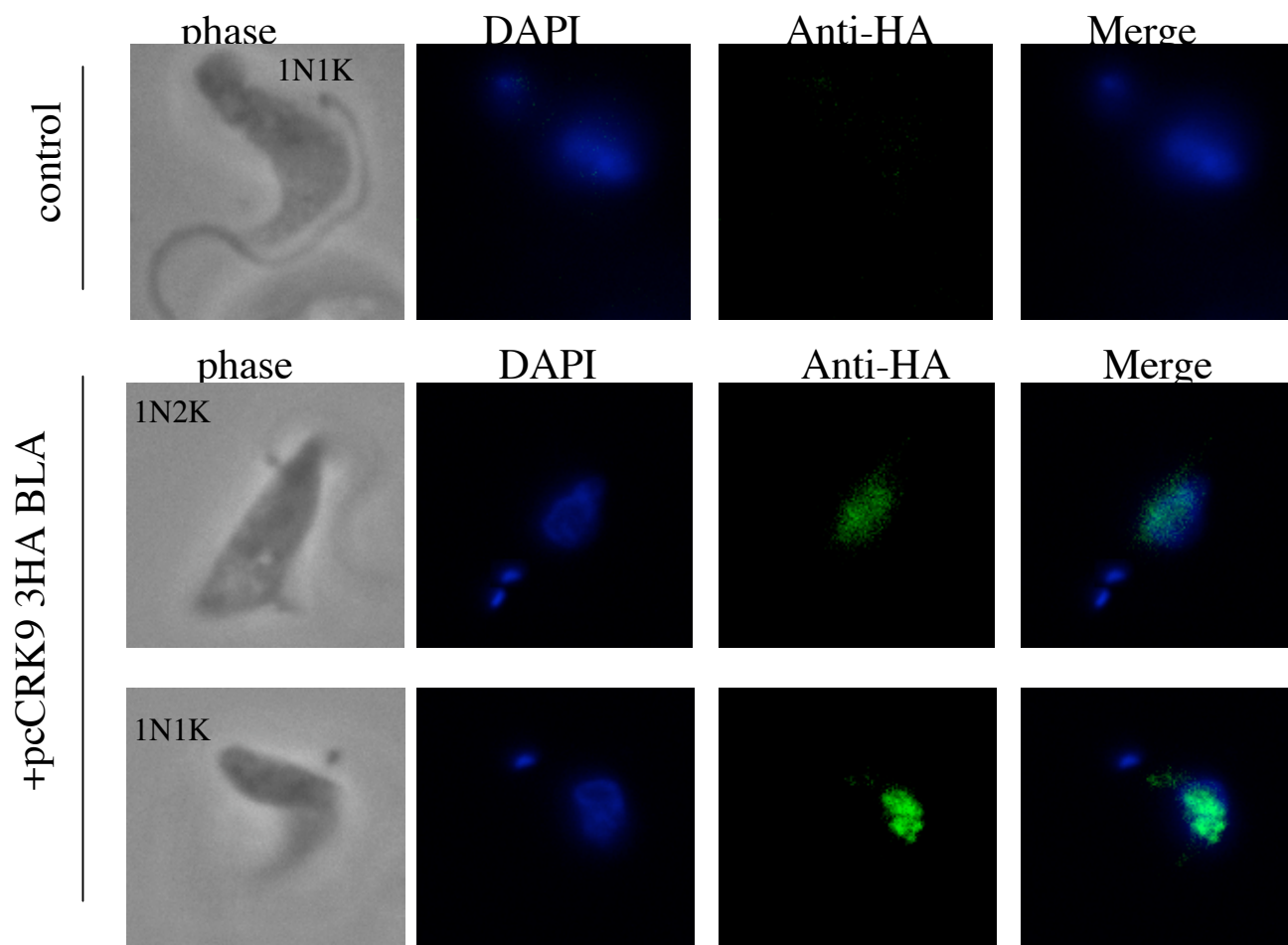

Supplement: Additional file 6 — Localization of CRK9 in bloodstream form cells. The data shows that CRK9 localizes to the nuclei of bloodstream form cells. The pc-CRK9-3HA-BLA plasmid was linearized, transfected into 90-13 bloodstream form T. brucei cells, and stable cell lines were selected using blasticidin (10 μg/ml) and cloned to express CRK9-3HA at the endogenous level. Transfected and control cells were analyzed by Western blotting for the HA tag as indicated (A). The blot were stripped and probed with anti-α-tubulin antibody to verify equal sample loading. Localization of CRK9 was determined by fixing and staining the transfected cells with a FITC-conjugated anti-HA antibody (B). DAPI was used to visualize the nucleus and kinetoplast and merged images were created using the imageJ software. [file 1471-2121-10-68-S6.PDF]
