# Supplementary material for: CRK9 contributes to regulation of mitosis and cytokinesis in the procyclic form of Trypanosoma brucei
Source: BMC Cell Biol. 2009 Sep 21;10:68. doi: 10.1186/1471-2121-10-68 (PMC2754446; doi:10.1186/1471-2121-10-68)
Supplement: Additional file 3 — Sequence alignment among the newly identified CRK homologues. The figure shows an alignment of the newly identified CRK homologues with the three CRKs 1, 2 and 3 whose functions have been demonstrated in previous studies, using the MacVector software. Residues important for catalysis, binding or the putative active site are indicated. A cartoon depicting the various domain structures of CRK9 compared with the well characterized CRK1-3 is shown in the top panel. [file 1471-2121-10-68-S3.PDF]

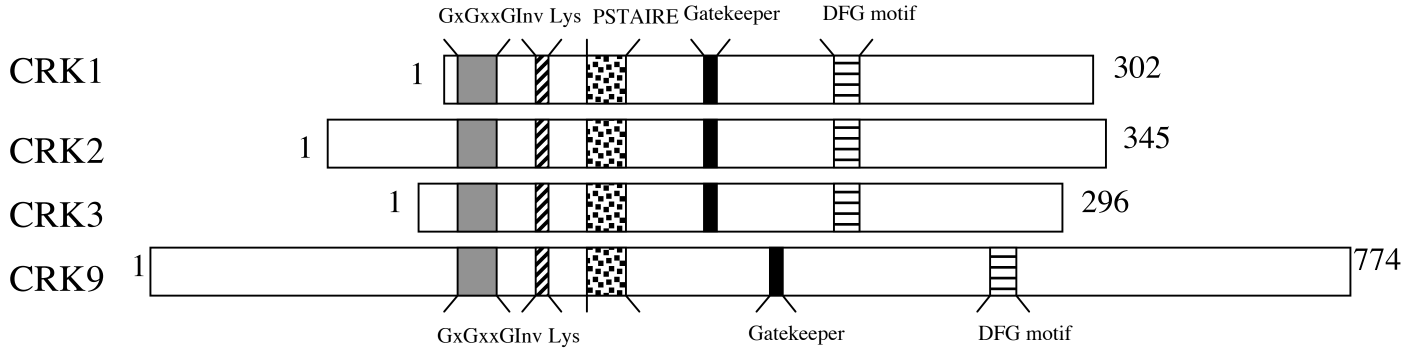
GxGxxG
Inv Lys
PSTAIRE
Gatekeeper
DFG motif
CRK1
1
302
CRK2
1
345
CRK3
1
296
CRK9
1
774
GxGxxG
Inv Lys
Gatekeeper
DFG motif

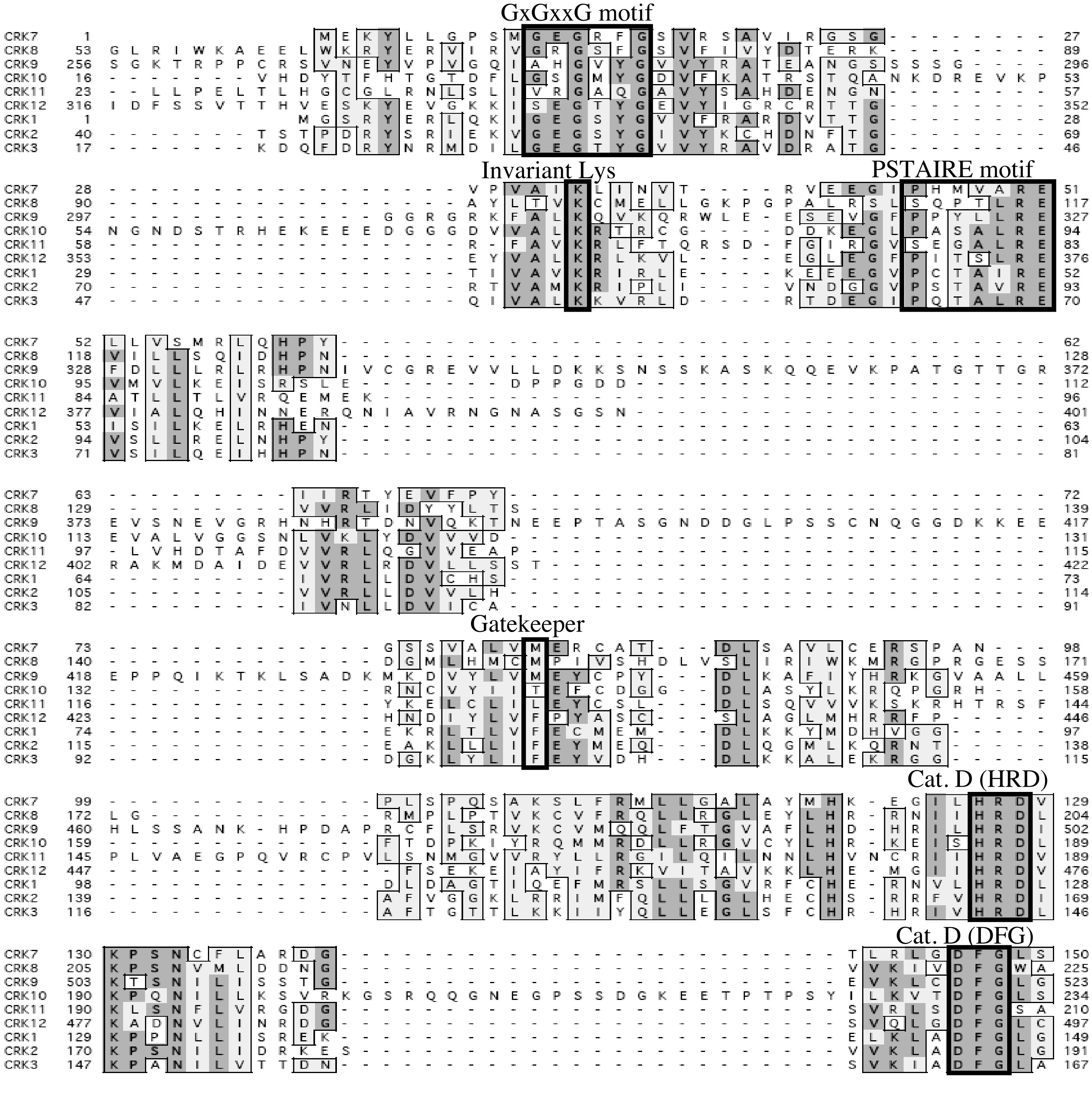
GxGxxG motif
Invariant Lys
PSTAIRE motif
Gatekeeper
Cat. D (HRD)
Cat. D (DFG)
CRK7
CRK8
CRK9
CRK10
CRK11
CRK12
CRK1
CRK2
CRK3
